# Endothelial Function and Postprandial Glucose Control in Response to Test-Meals Containing Herbs and Spices in Adults With Overweight/Obesity

**DOI:** 10.3389/fnut.2022.811433

**Published:** 2022-02-22

**Authors:** Yudai Huang, Meng-Fu Tsai, Rajrajeshwari Sunil Thorat, Di Xiao, Xuhuiqun Zhang, Amandeep K. Sandhu, Indika Edirisinghe, Britt M. Burton-Freeman

**Affiliations:** Department of Food Science Nutrition, Center for Nutrition Research and Institute for Food Safety and Health, Illinois Institute of Technology, Chicago, IL, United States

**Keywords:** herbs, spices, flow-mediated dilation (FMD), obesity, meal tolerance test, metabolic

## Abstract

**Objectives:**

Herbs and Spices (H/S) contain bioactive compounds with purported health benefits. This study investigated the effect of H/S intake on indicators of vascular and metabolic health over 24 h using a test-meal challenge paradigm in adults with overweight or obesity.

**Methods:**

In a randomized, single-blinded, 4-arm, 24 h, multi-sampling, crossover clinical trial, adults (*n* = 25) aged 36.6 ± 3.1 years with BMI 28.5 ± 0.6 kg/m^2^ (mean ± SEM) consumed a high-fat, high-carbohydrate challenge meal (~810 kcal) with salt/pepper only (control) or control with one of three different H/S combinations: Italian herb (rosemary, basil, thyme, oregano, and parsley), cinnamon, or pumpkin pie spice mix (cinnamon, ginger, nutmeg, and allspice) on four separate visits at least 3 days apart. Meals provided 35% of subjects' energy to maintain weight and ~1 g H/S per 135 kcal of the meal. Flow-mediated dilation (FMD) and blood samples were collected at 0, 1, 2, 4, 5.5, 7, and 24 h for endpoint analysis (additional blood draw at 0.5 h for insulin/glucose). Mixed-model analysis of repeated measures via PROC MIXED PC-SAS 9.4 was performed on the primary outcome (FMD) and secondary outcome variables. This study was registered at ClinicalTrials.gov (NCT03926442).

**Results:**

Italian herb and pumpkin spice meals significantly increased %FMD at 24 h compared to the control meal (*P* = 0.048 and *P* = 0.027, respectively). The cinnamon meal reduced postprandial glycemia (Δ) compared to control (*P* = 0.01), and pumpkin pie spice mix and cinnamon meals reduced postprandial insulin at 0.5 h compared to the control meal (*P* = 0.01 and *P* = 0.04, respectively). IL-6 and triglycerides increased in response to all meals (Time, *P* < 0.0001) but were not significantly different between meals.

**Conclusions:**

The test-meal challenge study design coupled with multiple sampling over 24 h provides insights into time-course bioactivity of H/S on vascular function and metabolic indices in overweight/obese adults.

**Clinical Trial Registration:**

ClinicalTrials.gov, identifier: NCT03926442.

## Introduction

Cardiovascular diseases (CVD) are the leading cause of death globally, killing ~18.6 million people in 2019 (1). Dietary patterns, smoking, physical inactivity, obesity, and endothelial dysfunction are risk factors for CVD. Endothelial dysfunction is an early-stage predictor of CVD events (1, 2) and is characterized by decreasing nitric oxide (NO) bioavailability and increasing inflammatory reactions in the blood vessel wall (2–4). Hyperglycemia, hypercholesterolemia, and hypertension are key contributors to endothelial dysfunction (5).

High-fat and high-carbohydrate (HFHC) meals, typical of modern-day eating patterns, induce postprandial spikes in glucose and insulin and prolong elevation of lipids, increasing oxidative stress and activating inflammatory genes (6–8). Oxidative stress in vascular cells decreases NO bioavailability and/or signaling, leading to impaired endothelial function, which promotes vascular cell proliferation and migration, inflammation and apoptosis, and extracellular matrix alterations, further contributing to endothelial dysfunction and the atherosclerotic disease process (3, 5). Obesity and diabetes exaggerate the risks caused by oxidative stress due to adiposity-associated chronic low-grade inflammation and prolonged metabolic recovery after a meal (9–11). Identifying practical and economically viable dietary strategies that alleviate acute and chronic physiological stresses is a strategic approach to prevent chronic disease development.

Herbs and spices (H/S) are common food seasoning ingredients with a long history in culinary and alternative medicine applications in eastern cultures (12). Only recently, H/S have been studied rigorously in western medicine for substantiating their use for disease risk reduction. H/S are rich dietary sources of bioactive phenolic terpenes and (poly)phenols, including flavonoids and phenolic acids (13–16). Common H/S, such as cinnamon, ginger, rosemary, thyme and oregano, may reduce disease risk by interacting with targets in inflammatory pathways, lowering low-density lipoprotein-cholesterol (LDL-c) concentrations, reducing platelet aggregation, and managing glucose metabolism (12, 14, 15, 17). Acute effects of H/S intake on postprandial vascular flexibility have been reported in previous studies up to 4 h after meal intake (18–20). Recent advances in (poly)phenol metabolism highlight microbial-derived metabolites from the gut microbiome that are available well beyond the typical postprandial period (21). Therefore, the present study aimed to characterize bioactivity of H/S on measures of endothelial function, metabolic and inflammation markers across an extended postprandial period, including an assessment 24 h after H/S intake in overweight/obese adults.

## Methods

### Study Design

This is a single-center randomized, single-blinded, 4-arm, 24 h, multi-sampling crossover clinical trial. Subjects who met eligibility criteria completed four study visits spanning a 24 h period/two days (day one for test meal and protocol-specified assessments and day two for follow up 24 h assessments). Each of the four study visits followed the same protocols, except the test meals differed based on each subject's randomization sequence. Day one of each study visit was separated by 3–10 days and lasted approximately 8.5 h and day two lasted ~1 h to conduct the 24 h follow-up assessments the next morning. Subjects were randomized to a test meal sequence on day one of their first study visit. Subjects were free-living with guidance on particular foods to avoid that may contain H/S similar to those being researched in the current study (e.g., Italian foods, cinnamon-flavored baked goods) or other foods/dietary supplements with published vascular or metabolic effects in humans (e.g., dark chocolate, all types of berries, tea, etc.). Subjects followed a standardized dinner meal protocol the evening before each visit to the Center for Nutrition Research (CNR) and recorded food intake for 3 days leading up to a visit to help remind subjects of study food restrictions. The study was conducted from April 2019 to March 2020 at the CNR at the Illinois Institute of Technology, Chicago, Illinois.

The study was approved by the Institutional Review Board of Illinois Institute of Technology Chicago, Illinois and registered at ClinicalTrials.gov (NCT03926442).

### Study Subjects

Adult men and women from the greater Chicagoland area were recruited through online surveys and screened for eligibility at the CNR. Eligibility criteria included adults aged 18–65 years with body mass index (BMI) of 25–35 kg/m^2^ who do not smoke or have documented history of chronic diseases (i.e., diabetes, hypertension, and cardiovascular disease, etc.), or take medications or dietary supplements that would interfere with the results of the study (i.e., lipid-lowering medication, anti-inflammatory drugs, fish oil, etc.). Subjects who screened with a fasting glucose > 125 mg/dL, or who were trained athletes or in training, or who exceeded four cups coffee/tea per day, or who worked overnight, or females who were pregnant or lactating were not eligible for participation. Premenopausal women were studied during the follicular phase of their menstrual cycle.

### Study Test Meal Intervention

A Taiwanese pancake (I-Mei Foods Co., Ltd., Taiwan) breakfast meal was the base HFHC test-meal served at every (first day) study visit. The meal contained salt and pepper alone (control) or salt/pepper with three different combinations of commercially-available H/S (McCormick, Hunt Valley, MD): Italian herb (mixture of rosemary, basil, thyme, oregano, and parsley in a 1:1:1:1 ratio), cinnamon, or pumpkin pie spice (mixture of cinnamon, ginger, nutmeg, and allspice in order of decreasing amount, ratio unknown). Meals were customized to provide 35% of subjects' energy to maintain weight (22) and ~1 g H/S per 135 kcal. The pancake used in the test-meal provided: (1) the desired vehicle to deliver the different flavors of the H/S interventions, (2) the carbohydrate and fat content to induce an acute oxidative-metabolic-inflammatory response typical of modern eating patterns, and (3) was easily adjusted to give a consistent dose of H/S per subject energy requirements. All ingredients were purchased from a local grocery store in Chicago, Illinois. Challenge meal composition is shown in Table 1.

**Table 1:** Composition of high-fat, high-carbohydrate (HFHC) breakfast test meals^a^ for postprandial study visits.

	**Control meal**	**Italian herb mix meal**	**Cinnamon meal**	**Pumpkin pie spice mix meal**
Energy (kcal)	830	829	825	830
Carbohydrate (g)	86.5	85.0	86.3	85.7
Total fat (g)	42.9	43.3	42.9	43.6
Saturate fat (g)	17.2	18.3	17.2	17.6
Protein (g)	25.4	24.7	25.4	25.6
Total fiber (g)	6.1	8.0	8.9	6.6
Cholesterol (mg)	488	480	488	488
Sodium (mg)	1,065	963	966	969
Italian herb (g)^b,c^	–	6	–	–
Cinnamon (g)^b^	–	–	6	-
Pumpkin pie spice mix (g)^b,d^	–	–	–	6
Pepper (g)^*b*^	0.6	0.6	0.6	0.6

a *Meals contained flaky scallion pancakes, eggs, cheddar cheese, butter, vegetable oil, and ketchup*.

b *Approximate weight; the amount of H/S in the meals is calculated to 1 g H/S per 135 kcal*.

c *Italian herb mix contains the same amount of rosemary, basil, thyme, oregano, and parsley*.

d *Pumpkin pie spice mix contains cinnamon, ginger, nutmeg and allspice, in decreasing order of amount*.

### Study Visits

**Figure 1:**
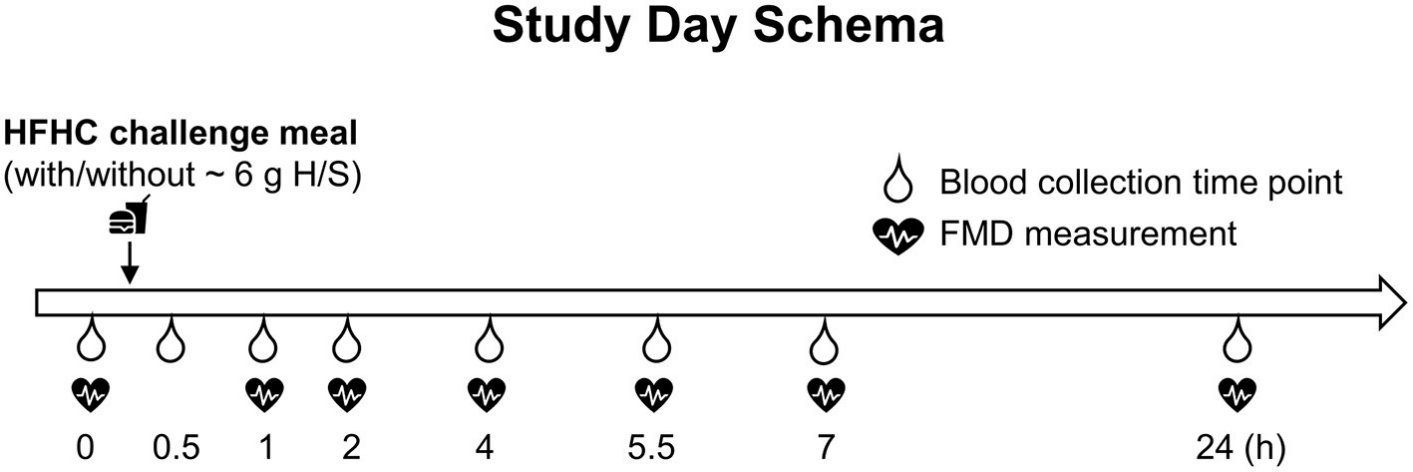
Seven hours of postprandial and 24 h follow up study visit schema. FMD, flow-mediated dilation; HFHC, high-fat and high-carbohydrate; H/S, herbs and spices.

Subjects arrived at the CNR in the fasting state (10–14 h before the appointment) and completed routine pre-study assessments, including compliance with fasting, adequate sleep, diet, medication/supplementation change, vitals, and anthropometric measurements. Thereafter, an intravenous catheter was placed into the subjects' non-dominant arm by a licensed health care practitioner and a baseline blood sample was collected (0 h). Based on the randomization sequence, subjects were then provided one of the four challenge test meals to finish within 20 min. Water was provided throughout the study day visits. After the 7 h blood collection, the catheter was removed and a standardized snack was provided. Subjects were evaluated for safety before leaving the CNR. Subjects consumed a standardized dinner meal and came back to the CNR the next morning fasted (10–14 h) for the 24 h assessments completing the study visit. Study visit schema is shown in Figure 1.

### Blood Collection and Analysis

Blood samples were collected at 0, 0.5, 1, 2, 4, 5.5, 7, and 24 h in vacutainer plastic blood collection tubes with ethylenediaminetetraacetic acid (EDTA; Thermo Fisher Scientific, Waltham, MA) and immediately placed on ice. To obtain plasma, blood samples were centrifuged for 15 min at 4°C at 453 *g*, and then aliquoted into individual vials and stored at −80°C until analysis.

Insulin, glucose, and triglyceride analysis were conducted on plasma samples at all time points using a RX Daytona automated clinical analyzer (Randox Laboratories, Crumlin, UK). Interleukin-6 (IL-6) was analyzed at 0, 2, 4, 5.5, 7, and 24 h using the Human IL-6 Quantikine High Sensitive ELISA Kit (R&D Systems, Minneapolis, MN). All analyses were performed according to manufactures' protocols with quality controls.

### Flow-Mediated Dilation

FMD was measured at 0, 1, 2, 4, 5.5, 7, and 24 h time points by a high-resolution B-mode ultrasound system (GE LOGIQ e, UK) following standardized methods recommended by the American College of Cardiology with minor modification used in our lab (23). Briefly, subjects rested in a dimmed-light, quiet, temperature-controlled room for 15 min prior to testing. The diameter of the brachial artery (mm) was measured above the elbow of the non-catheter arm for pre-occlusion baseline values. Post-occlusion brachial artery diameter was measured every 5 s for 2 min immediately after 5 min occlusion with blood pressure cuff inflated to 220 mmHg for 5 min below the elbow. FMD is expressed as the percent change in arterial diameter between post- and pre-occlusion compared with the diameter at pre-occlusion baseline (FMD%). A single trained researcher performed all FMD measurements to maintain consistency.

### Statistical Analysis

Randomization schedules and outcome variables were constructed/analyzed using PC-SAS 9.4 (SAS Institute, Inc., Cary, North Carolina). Randomization was based on Williams design for crossover studies using SAS PROC Plan (24). Subject characteristics were analyzed and tabulated using descriptive statistics. Mixed-model analysis of repeated measure via PROC MIXED was used to test the main effects of the test meal intervention, time, and their interaction (meal by time) on primary and secondary endpoints using data from the modified intent-to-treat sample population (mITT). The mITT was defined as sample data from subjects who were randomized and completed at least one study visit (*n* = 25) less the blood data unavailable/removed for two subjects due to catheter failure and diabetes level fasting glucose (25). The primary endpoint was FMD and secondary endpoints for analysis were glucose, insulin, triglycerides, and IL-6. Shapiro-Wilks test was used to evaluate the normality of endpoint data distributions. Log_10_ transformation was applied on datasets not conforming to normal distribution before analysis, including FMD, insulin, TG, and IL-6. Delta differences in postprandial metabolic responses were evaluated by normalizing data to each subject's own baseline (0 h) (i.e., subtracting each subject's baseline values from their postprandial values after each meal). Covariates, such as randomization sequence, age, BMI, gender, race, were tested and included in final models when significant. Kenward-Roger correction and the method of restricted maximum likelihood were used in all Mixed Models (26–28). Type 3 test of fixed effects and tests of effect slices were used to evaluate main and simple effects, respectively (29).

*Post-hoc* analyses were performed when significance was achieved in the main or simple effects model. Multiple comparisons were corrected using Tukey-Kramer or Dunnett-Hsu adjustment in mixed model procedures. Data are presented as means ± standard error of the means (SEM). *P* < 0.05 was considered statistically significant for a null hypothesis of no difference between study test meals.

## Results

### Subject Demographics

**Figure 2:**
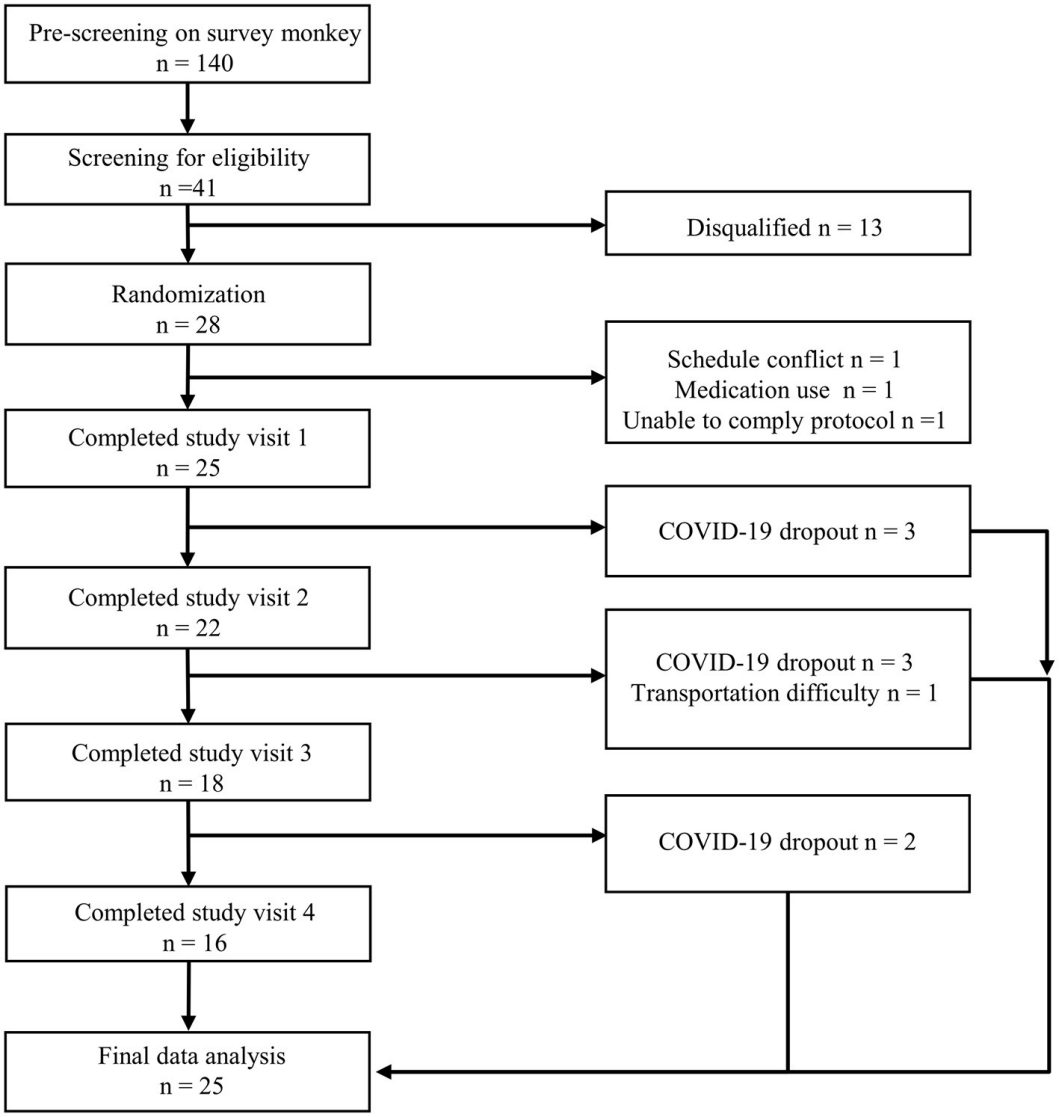
Consolidated Standards of Reporting Trials (CONSORT) flow diagram of the study.

One hundred and forty subjects completed online survey questionnaires, and 41 were qualified for pre-screening at the CNR. Twenty-eight subjects were eligible and enrolled in the study. Three subjects withdrew prior to randomization due to schedule conflict, medication usage, or unable to comply with the protocol. The evaluable data set was derived from the modified intent-to-treat sample population (*n* = 25, see Statistical Analysis Section). Nine of the 25 subjects completed at least one study day visit but were unable to complete the entire study due to the COVID-19 pandemic (Figure 2). Thirteen men and 12 women aged 36.6 ± 3.1 years with a mean BMI of 28.5 ± 0.6 kg/m^2^ participated in the research. Demographic characteristics are shown in Table 2.

**Table 2:** Subject demographic information at the screening visit.

**Variable**	**All (*n* = 25)**	**Aged 18–40 (*n* = 16)**	**Aged 41–65 (*n* = 9)**
Male: Female (n)	13:12	9:7	4:5
Race (AA:ASIAN:CAU:NA)	8:9:7:1	3:9:4:0	5:0:3:1
Age (y)	36.6 ± 3.1	25.9 ± 1.1	55.6 ± 2.4
BMI (kg/m^2^)	28.5 ± 0.6	27.8 ± 0.6	29.8 ± 1.1

*AA, African American; CAU, Caucasian; NA, Native American. Data are means ± standard error of the mean (SEM)*.

### Postprandial Changes in FMD, *n* = 25

Percent FMD significantly increased over time after meals with H/S compared to control (meal by time interaction, *P* = 0.04, Table 3 and Supplementary Figure 1) with main differences observed at 24 h (*P* = 0.02, Figure 3). After an initial dip, %FMD began to increase about 4 h after the Italian herb meal and 7 h after the cinnamon meal (Table 3 and Supplementary Figure 1); however, H/S-related FMD was not significantly higher than control until 24 h (Italian herb 8.1 ± 0.7% FMD, and pumpkin pie spice mix 7.9 ± 0.6% FMD test meals vs. control 6.5 ± 0.8% FMD, *P* = 0.048 and *P* = 0.027, respectively, Figure 3 and Table 3). The cinnamon test meal also increased %FMD (8.1 ± 0.9 FMD %) at 24 h, but not significantly compared to control (*P* = 0.06).

**Table 3:** Flow-mediated dilation (FMD) over 24 h^a^.

**FMD (%) Time-point**							
**Test meals**	**0 h**	**1 h**	**2 h**	**4 h**	**5.5 h**	**7 h**	**24 h**
Control	6.5 ± 0.7	7.1 ± 0.8	7.2 ± 0.9	7.3 ± 1.0	6.8 ± 0.7	6.5 ± 1.0	6.5 ± 0.8
Italian herb	6.5 ± 0.7	6.4 ± 0.7	6.0 ± 0.6	8.2 ± 0.9	8.0 ± 0.7	7.5 ± 0.6	8.1 ± 0.7^*^
Cinnamon	6.5 ± 0.8	5.1 ± 0.5	7.4 ± 0.6	7.3 ± 1.1	6.5 ± 0.9	7.8 ± 0.8	8.1 ± 0.9^#^
Pumpkin pie	7.4 ± 0.6	6.2 ± 0.7	6.0 ± 0.7	7.3 ± 0.7	7.0 ± 0.7	6.4 ± 0.6	7.9 ± 0.6^*^

a *Main effect of meal (P = 0.26), time (P = 0.17), age (P = 0.02), and significant meal by time interaction (P = 0.04). Data are means ± standard error of mean, n = 25. %FMD, percentage change in flow-mediated dilation. Data were log-transformed prior to statistical analysis. PROC MIXED (SAS Institute, Inc., Cary, North Carolina) was used for repeated measures. Dunnett-Hsu adjustment was used in the model. Significantly different from control*,

* *P < 0.05*,

# *P = 0.06*.

**Figure 3:**
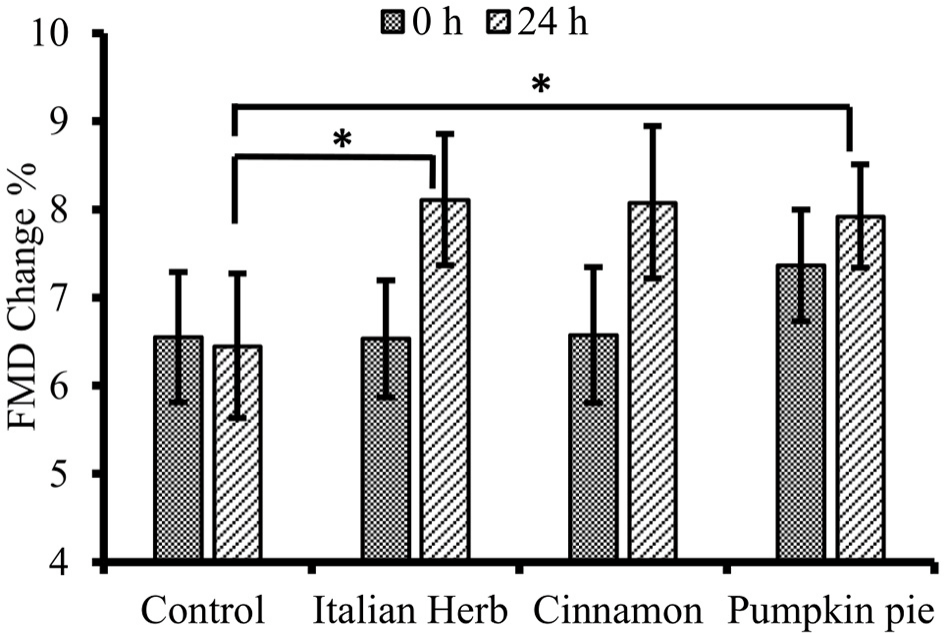
Fasting/baseline flow mediated dilation (FMD) pre-meal (0 h) vs. 24 h post-challenge meal (*n* = 25). Data are means ± standard error of mean (SEM). Data were log-transformed prior to statistical analysis. PROC MIXED (SAS Institute, Inc., Cary, North Carolina) was used for repeated measures. Significant meal by time interaction (*P* = 0.04) with main difference at 24 h (*P* = 0.02). *Significantly different from control, *P* < 0.05.

### Postprandial Changes in Metabolic and IL-6 Responses, *n* = 23

#### Glucose

**Figure 4:**
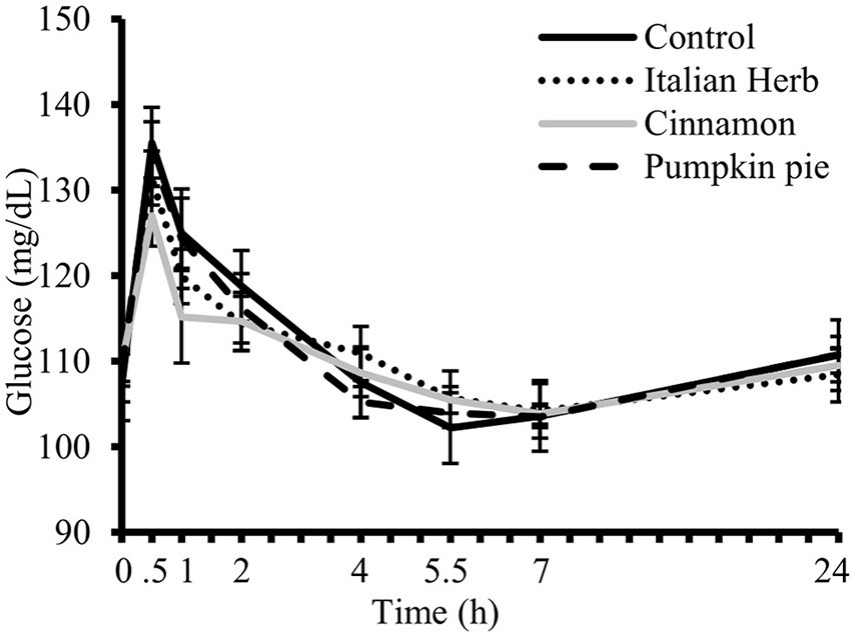
Postprandial plasma glucose concentrations over 7 h after consuming high-fat and high-carbohydrate (HFHC) challenge test meals with and without addition herbs and spices (H/S) followed by fasting 24 h plasma glucose concentration (*n* = 23). Main effect of meal (*P* = 0.17), time (*P* < 0.0001). Data are means ± standard error of mean. PROC MIXED (SAS Institute, Inc., Cary, North Carolina) was used for repeated measures.

Plasma glucose concentrations increased after intake of study test meals peaking at 0.5 h and returning to baseline by 2 h (Time effect, *P* < 0.0001, Figure 4). The main effect of the meal on glucose (*P* = 0.02, Table 4) responses showed significant attenuation of postprandial (Δ) glycemia when cinnamon was added to the meal compared to control (*P* = 0.01, Table 4).

**Table 4:** Postprandial changes (Δ) in plasma glucose, insulin, triglycerides, IL-6^a^.

	**Test meals**	**0 h**	**0.5 h**	**1 h**	**2 h**	**4 h**	**5.5 h**	**7 h**	**24 h**	***P*-value meal**	***P*-value time**	***P*-value meal × time**
ΔGlucose (mg/dL)	Control	0	28.34 ± 3.8	17.77 ± 4.4	11.61 ± 3.3	0.34 ± 2.2	−5.00 ± 2.1	−3.60 ± 1.9	3.50 ± 1.1	0.0209	<0.0001	0.6193
	Italian herb	0	22.98 ± 3.0	11.49 ± 5.5	5.97 ± 3.1	2.48 ± 2.3	−2.75 ± 2.1	−4.22 ± 1.8	−0.02 ± 1.2			
	Cinnamon^†^	0	17.26 ± 2.9	5.47 ± 5.0	4.95 ± 3.0	−1.06 ± 2.8	−4.25 ± 2.1	−5.90 ± 1.6	−0.23 ± 0.7			
	Pumpkin pie	0	25.26 ± 3.9	15.35 ± 5.8	7.60 ± 4.1	−2.98 ± 2.3	−4.65 ± 2.0	−5.48 ± 1.6	1.79 ± 1.8			
ΔInsulin^b^ (uIU/mL)	Control	0	66.39 ± 7.6	63.90 ± 7.1	37.75 ± 6.2	5.24 ± 1.5	−0.86 ± 0.6	−0.43 ± 0.5	1.10 ± 0.5	0.1447	<0.0001	0.0034
	Italian Herb	0	59.19 ± 5.0	43.99 ± 4.2^#^	31.41 ± 2.6	7.79 ± 1.3	0.39 ± 0.6	−0.89 ± 0.5	2.37 ± 0.8			
	Cinnamon	0	49.19 ± 5.6^*^	56.13 ± 8.3	35.68 ± 3.7	9.14 ± 3.0	2.04 ± 1.9	−0.84 ± 0.6	0.93 ± 0.7			
	Pumpkin pie	0	46.90 ± 6.2^*^	49.97 ± 5.1	36.47 ± 6.4	7.28 ± 2.2	0.21 ± 0.6	−1.50 ± 0.5	1.01 ± 0.8			
ΔTG^b^ (mg/dL)	Control	0	8.67 ± 3.2	40.72 ± 10.0	68.41 ± 10.3	45.08 ± 9.8	12.65 ± 6.1	−4.61 ± 4.9	−5.87 ± 4.1	0.3389	<0.0001	0.6846
	Italian Herb	0	9.56 ± 4.0	37.33 ± 9.9	64.19 ± 13.3	62.58 ± 11.7	28.10 ± 8.3	7.49 ± 6.1	−8.03 ± 5.6			
	Cinnamon	0	3.18 ± 3.9	23.63 ± 5.6	53.85 ± 6.1	61.07 ± 11.4	23.11 ± 6.1	2.06 ± 6.0	−4.88 ± 7.3			
	Pumpkin pie	0	10.86 ± 3.6	31.61 ± 5.6	69.72 ± 9.6	66.05 ± 13.0	26.56 ± 7.6	−0.82 ± 5.1	−8.29 ± 6.0			
ΔIL-6^b^ (pmol/L)	Control	0	–	–	0.00 ± 0.2	1.95 ± 0.7	3.90 ± 1.1	3.39 ± 1.0	0.18 ± 0.3	0.3795	<0.0001	0.709 7
	Italian herb	0	–	–	0.39 ± 0.2	1.97 ± 0.7	3.27 ± 0.7	3.78 ± 0.6	0.12 ± 0.3			
	Cinnamon	0	–	–	0.37 ± 0.3	1.47 ± 0.6	2.08 ± 0.6	3.47 ± 0.9	0.35 ± 0.3			
	Pumpkin pie	0	–	–	0.20 ± 0.2	1.33 ± 0.5	2.70 ± 0.8	4.53 ± 1.0	1.02 ± 0.6			

a *Data are means ± standard error of mean, n = 23. IL-6, interleukin-6; TG, triglycerides. PROC MIXED (SAS Institute, Inc., Cary, North Carolina) was used for repeated measures. Tukey-Kramer adjustment was used in the model. Significant difference from control group*;

* *P < 0.05*,

# *P = 0.056. Significant meal effect compared to the control*

† *P = 0.01*.

b *Age by time interaction was included in the final model*.

#### Insulin

**Figure 5:**
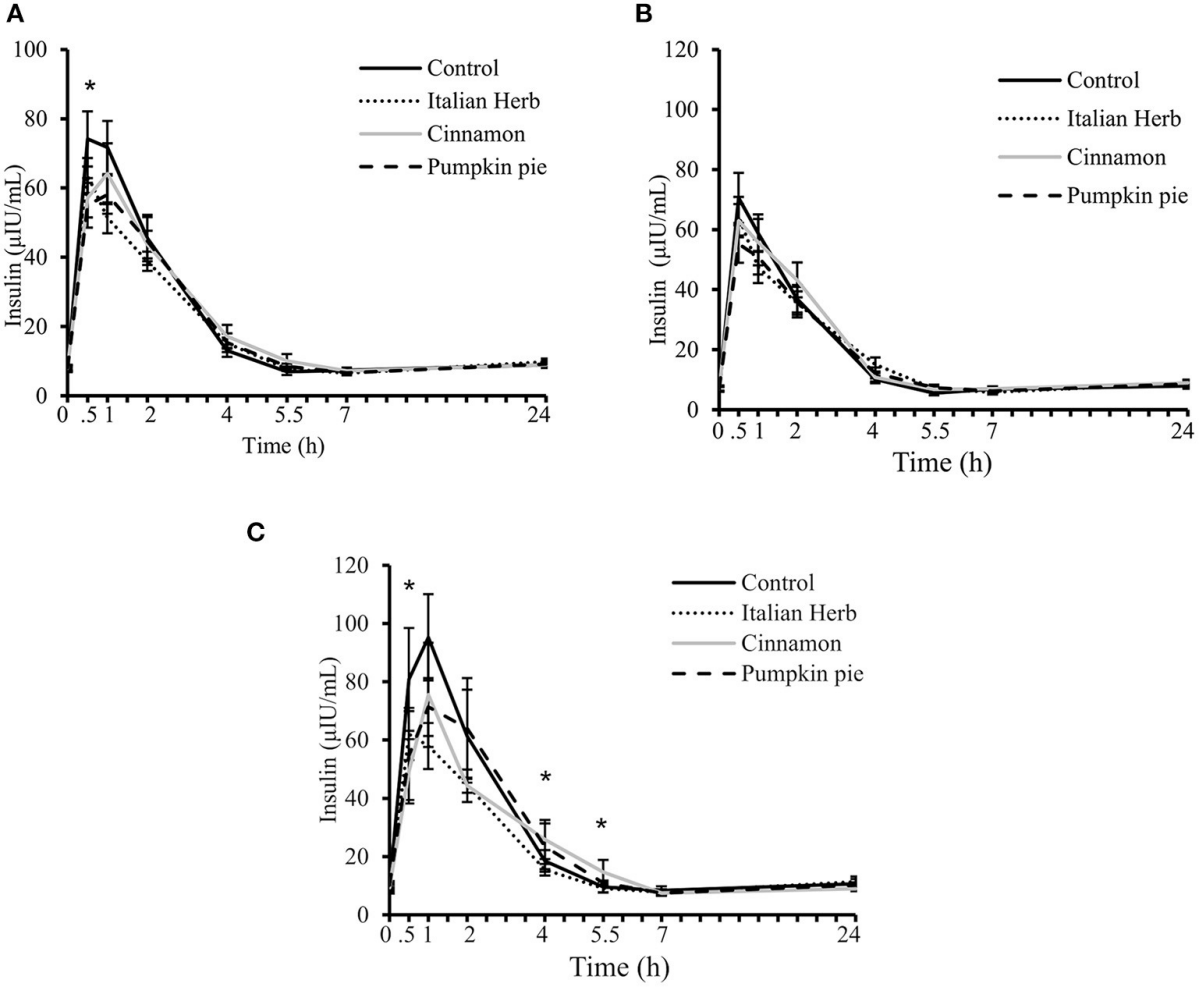
Postprandial plasma insulin concentrations over 7 h after consuming high-fat and high-carbohydrate (HFHC) challenge test meals with and without addition herbs and spices (H/S) followed by fasting 24-h plasma insulin concentration in **(A)** All evaluable subjects (*n* = 23); **(B)** subjects with age <40 years (*n* = 15); **(C)** subjects with age ≥40 years (*n* = 8). Main effect of meal (*P* = 0.12), time (*P* <0.0001), meal by time interaction (*P* = 0.04), and meal by time by age interaction (*P* = 0.001). Data are means ± standard error of mean. Data were log-transformed prior to statistical analysis. PROC MIXED (SAS Institute, Inc., Cary, North Carolina) was used for repeated measures. *Significant meal effect at the time point, *P* <0.05.

Plasma insulin significantly increased after intake of all meals peaking at 0.5 or 1 h depending on the study test meal and returning to baseline by 5.5 h (time effect *P* <0.0001, time by meal interaction *P* = 0.04, Figure 5A). Postprandial (Δ) time by meal interaction was also significant (*P* = 0.003, Table 4). Changes in postprandial insulin were significantly different between the control meal (Δ 66.39 ± 7.6 uIU/mL) and the cinnamon and pumpkin spice meals at 0.5 h (Δ 49.19 ± 5.6 and Δ 46.90 ± 6.2 uIU/mL, respectively, *P* <0.05 both, Table 4); and, after the control meal vs. Italian herb at 1 h (Δ 63.90 ± 7.1 vs. Δ 43.99 ± 4.2 uIU/mL, *P* = 0.056, Table 4). Age by time interaction was significant and included in the final model, suggesting individuals over 40 years may manage hyperinsulinemia better by adding H/S to their daily meals (Figures 5B,C).

#### Triglycerides (TG)

Plasma TG significantly increased after consuming all study test meals and returned to baseline by 7 h (time effect, *P* <0.0001). Mean TG concentrations were higher after the Italian herb meal compared to control (*P* = 0.04), but not after controlling for each subject's own baseline. Changes in postprandial responses (Δ) were not significantly different among test meals (*P* > 0.05).

#### Interleukin (IL)-6

Plasma IL-6 significantly increased after consuming all meals and did not return to baseline until measured again at the 24 h time point (Time effect, *P* <0.0001). No difference in IL-6 responses, including peak concentrations were observed between meals (Table 4).

## Discussion

The current study used a multi-sampling test-meal study design to characterize the time-course of H/S biological action on measures of endothelial function, postprandial metabolic control, and inflammation in adults with overweight/obesity. Endpoints of interest included meal-induced changes in FMD, insulin, glucose, triglycerides, and IL-6. The results demonstrated variable FMD in response to H/S containing meals during the initial 7 h period with patterns of increased postprandial vaso-relaxation at ~4 and 7 h after the Italian herb and cinnamon containing test meals, respectively; however, it was not until 24 h that H/S test meals produced significantly increased FMD compared to the control meal. In addition, we observed significant attenuation of meal-induced glucose and insulin responses when cinnamon was added to the test meal, whereas pumpkin pie spice mix and Italian herb attenuated meal-induced insulin responses, but not glucose, compared to the control meal.

It is well-documented that modern-day eating patterns promote metabolic imbalance and oxidative stress that over time leads to cellular/tissue/organ dysfunction and eventually disease (8). Excessive reactive oxygen and nitrogen species (ROS and RNS) in endothelial cells decrease NO bioavailability/signaling contributing to endothelial dysfunction and CVD development (30). Dietary strategies to increase antioxidant defenses, particularly in endothelial cells, are practical for vascular health. H/S contain compounds with antioxidant properties revealed through various studies examining their free radical scavenging capability *in vitro* (31), in the kitchen and *in vivo* (32). Li et al. (32) reported a 71% decrease in malondialdehyde (MDA), a lipid peroxidation product, in a hamburger cooked with spices (11.25 g spice mix including cloves, cinnamon, oregano, rosemary, ginger, black pepper, paprika and garlic powder) compared to control burgers cooked without spices. Further, they reported decreased urine MDA and a trend for decreased plasma MDA in healthy subjects consuming the burgers with the aforementioned 11.25 g spice mix compared to control burgers without spices (*n* = 11, age 31.3 ± 2.5 years, BMI 25.6 ± 1.4 kg/m^2^) (32). In a subsequent study, the same group reported higher peripheral arterial tonometry (PAT, a measure of endothelial function) scores 2 h after eating a burger with spice mix vs. without spices in male subjects with type 2 diabetes mellitus (T2DM, *n* = 18, 60.9 ± 7.5 years, 31.9 ± 6.1 kg/m^2^). The PAT results were supported by increased urinary nitrate/nitrite concentration after 6 h, suggesting enhancement of NO bioavailability (19). Others have also reported enhanced vaso-relaxation with spice interventions. Nakayama et al. (18) reported increased FMD at 1 h after consumption of a curry meal containing 23.5 g spice blend (onion, turmeric, garlic, ginger, coriander, clove, cumin, and red pepper) compared to control meal without spices in healthy male subjects (*n* = 24, 45 ± 9 years, 23.7 ± 2.7 kg/m^2^). Peterson et al. (20) reported increased FMD at 4 h following a decline at 2 h after consuming 6 g of spice mix (basil, bay leaf, black pepper, cinnamon, coriander, cumin, ginger, oregano, parsley, red pepper, rosemary, thyme, and turmeric) incorporated in a high-saturated fat and high-carbohydrate meal in male subjects with central adiposity (*n* = 13, 52 ± 9 years, 29.9 ± 3.1 kg/m^2^, waist circumference 102.2 ± 8.9 cm). Similar to Petersen et al. (20), but not Li et al. (19) and Nakyama et al. (18) results, we observed a non-significant drop in FMD during the first couple hours of H/S intake before seeing a rebound and increase in FMD later in the postprandial and post-absorptive period. A hormetic effect may be one explanation (33), or differences in dose/bioavailability of antioxidant or vascular modulating H/S compounds in the first 2 h may be another explanation. Our results indicate increases in FMD starting around 4 h after intake of Italian herb- and 7 h after intake of cinnamon-containing HFHC meals. However, 24 h later FMD was significantly greater after H/S containing test meals compared to the control meal. H/S are concentrated sources of (poly)phenols, many of which are known to depend on the gut microbiota for transformation and absorption. Work in our lab and others have demonstrated (poly)phenols from different food sources vary in their absorption and clearance patterns, with some metabolites peaking early in the postprandial period (1–2 h), other metabolites later (4–6 h) and still others at 24 h or later (21, 34, 35). Pharmacokinetic studies characterizing H/S metabolites after intake may help explain the observed effects in this study, especially metabolites that become available to vascular beds later in the post-absorptive period.

H/S intake is associated with improved metabolic health demonstrated in *in vitro* and *in vivo* animal models and some human studies (36). Supplementation with ginger and cinnamon combination (15% diet w/w) for 30 days (37) or ginger, cinnamon and turmeric combination (300 mg/kg/day) for 6 weeks (38) improved lipids and glycemic control and reduced markers of oxidative stress in streptozotocin (STZ)-induced diabetic rats. Similarly, in a high-fructose diet fed animal model, adding the cinnamon extract to rodents' drinking water (300 mg/kg/day) for 3 weeks prevented diet-induced insulin resistance by enhancing insulin signaling possibly through NO pathway in skeletal muscle cells (39). Extracts of rosemary, basil and other herbs have also shown anti-diabetic effects in chemically-induced diabetic animal models demonstrating restoration of β cell function and insulin secretion and/or improving peripheral glucose uptake mechanisms involving insulin-dependent and -independent mechanisms (40). *In vitro* studies have examined preabsorptive mechanisms of H/S bioactivity and found potent inhibition of α-glucosidase and α-amylase enzymes involved in carbohydrate digestion and absorption (36, 41). Hence, H/S may act on a number of disease targets relevant for improving glucose homeostasis and metabolic health (36).

Compared to the pre-clinical literature, fewer studies have examined the metabolic effects of H/S consumption in humans. Medagama et al. (42) reviewed the evidence for cinnamon and reported favorable effects of cinnamon on glucose and lipids homeostasis in individuals with T2DM. Other studies report the glucose and lipid-lowering effect of ginger and rosemary supplementation in people with T2DM (43–46). Overall, data are accumulating to support metabolic benefits of H/S in humans with T2DM, although very little is known about H/S intake in at-risk individuals as a preventative strategy in disease development/transition (47–49). Our study reported on the acute postprandial responses of H/S intake in adults with overweight/obesity, a major risk factor for diabetes. Our research provides evidence that cinnamon attenuates postprandial glucose excursions as well as reduces the amount of insulin needed to manage postprandial glycemia. We also report attenuated insulin after eating HFHC test meals with Italian herb and pumpkin spice mix, an effect that may be accentuated by age, requiring further research. In contrast, Petersen et al. did not observe any effect on postprandial glucose or insulin by adding H/S to test meals. Neutral findings may be reflective of lower mean fasting glucose (97.3 ± 11.5 vs. 108.6 ± 1.0 mg/dL, Petersen et al. vs. the present study) of their group or suggest a dose requirement of specific H/S to elicit glucose-lowering or insulin-sensitizing responses. Individual H/S levels were lower in their mix compared to ours. For example, each of the Italian herbs in our study was delivered at ~1.2 g/meal, whereas each of the Italian herbs in Petersen et al. was delivered at <0.6 g/meal (20). Future research to reveal the full spectrum of benefits of culinary H/S to (re)establish and maintain metabolic flexibility will require study designs that systematically test their dose-response bio-efficacy in different stages of disease development and in different age groups. This approach takes into account the varied pathophysiological processes leading to diabetes and is consistent with acquiring knowledge that supports nutrition for precision health: *providing the right diet for the right person at the right time*.

The study has strengths and limitations. Ending the study early due to the pandemic was a limitation resulting in a modified completer/intent-to-treat analysis. However, the research had multiple strengths in its design and standardization of protocols for managing variability among study subjects and increasing the precision of the research. Standardized dinner meals were provided to study subjects the night before each study visit day along with study preparation instructions for consistency between visits. Additionally, H/S doses were standardized based on subjects' body weight and test-meal calorie intake to deliver ~1 g H/S per 135 kcal with a total of ~6.0 ± 0.5 g/meal. The amount of H/S tested by us and others (18, 19) may be considered too much for some people's liking and a limitation of the study; however, based on feedback from subjects in our study, many commented the H/S level was acceptable. The average daily consumption of H/S is between 0.5 and 4.4 g/day per person in the world, yet changing dietary habits and preference for ethnic and spicy foods in North America may suggest the preferred amount of H/S is increasing (50). This is an area for future research and possibly an area for attention by the food industry.

In summary, the present research provides new findings of H/S intake on endothelial function and the potential role of common culinary H/S in managing postprandial metabolic responses to HFHC meals. To the best of our knowledge, this is the first study to characterize the bioactivity of H/S incorporated into a test meal on vascular reactivity as measured by FMD over 24 h. Future research will be required to identify specific mechanisms of action and the H/S phytochemical metabolites responsible for study outcomes.

## Data Availability Statement

The original contributions presented in the study are included in the article/Supplementary Material, further inquiries can be directed to the corresponding author/s.

## Ethics Statement

The studies involving human participants were reviewed and approved by the Institutional Review Board of Illinois Institute of Technology Chicago, Illinois. The patients/participants provided their written informed consent to participate in this study.

## Author Contributions

BB-F, IE, and AS designed the study. M-FT, RT, and DX conducted the research and analysis of bio-specimen samples and FMD. XZ and BB-F performed statistical analysis. YH and BB-F wrote the manuscript. All authors read, edited, and approved the final manuscript.

## Funding

This project was supported by a gift from McCormick Science Institute and various donor funds.

## Conflict of Interest

BB-F serves on the advisory board for McCormick Science Institute. The remaining authors declare that the research was conducted in the absence of any commercial or financial relationships that could be construed as a potential conflict of interest.

## Publisher's Note

All claims expressed in this article are solely those of the authors and do not necessarily represent those of their affiliated organizations, or those of the publisher, the editors and the reviewers. Any product that may be evaluated in this article, or claim that may be made by its manufacturer, is not guaranteed or endorsed by the publisher.

## Supplementary Material

Supplementary Figure 1 Postprandial flow-mediated dilation (FMD) over 7 h after consuming high-fat and high carbohydrate (HFHC) challenge test meals with and without addition herbs and spices (H/S) followed by fasting 24 h FMD (*n* = 25). Main effect of meal (*P* = 0.26), time (*P* = 0.17), age (P = 0.02), and significant meal by time interaction (*P* = 0.04). Data are means ± standard error of mean, *n* = 25. %FMD, percentage change in flow-mediated dilation. Data were log-transformed prior to statistical analysis. PROC MIXED (SAS Institute, Inc., Cary, North Carolina) was used for repeated measures and Dunnett-Hsu for *post-hoc* comparisons.

## Abbreviations

BMI: body mass index
CNR: Center for Nutrition Research
CVD: cardiovascular disease
EDTA: ethylenediaminetetraacetic acid
FMD: flow-mediated dilation
HFHC, high-fat: high-carbohydrate
H/S: herbs and spices
IL-6: interleukin-6
LDL-c: low-density lipoprotein-cholesterol
MDA: malondialdehyde
mITT: modified intent-to-treat
NO: nitric oxide
PAT: peripheral arterial tonometry
RNS: reactive nitrogen species
ROS: reactive oxygen species
SEM: standard error of the means
STZ: streptozotocin
TG: triglycerides
T2DM: type 2 diabetes milletus.
