# Supplementary figures and images for: Endothelial Function and Postprandial Glucose Control in Response to Test-Meals Containing Herbs and Spices in Adults With Overweight/Obesity
Source: Front Nutr. 2022 Feb 22;9:811433. doi: 10.3389/fnut.2022.811433 (PMC8902252; doi:10.3389/fnut.2022.811433)

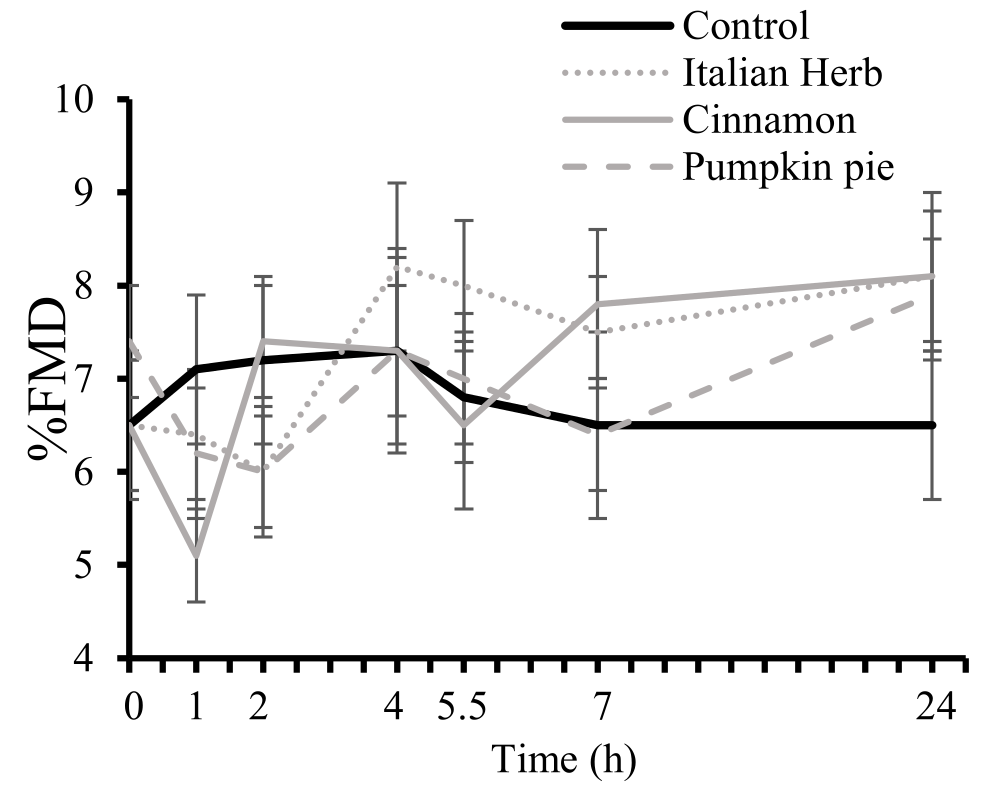

Supplement: Supplementary Figure 1 — Postprandial flow-mediated dilation (FMD) over 7 h after consuming high-fat and high carbohydrate (HFHC) challenge test meals with and without addition herbs and spices (H/S) followed by fasting 24 h FMD (n = 25). Main effect of meal (P = 0.26), time (P = 0.17), age (P = 0.02), and significant meal by time interaction (P = 0.04). Data are means ± standard error of mean, n = 25. %FMD, percentage change in flow-mediated dilation. Data were log-transformed prior to statistical analysis. PROC MIXED (SAS Institute, Inc., Cary, North Carolina) was used for repeated measures and Dunnett-Hsu for post-hoc comparisons. [file Image_1.TIF]
